# Alternative Interventions to Prevent Oxidative Damage following Ischemia/Reperfusion

**DOI:** 10.1155/2016/7190943

**Published:** 2016-12-28

**Authors:** Simón Quetzalcoatl Rodríguez-Lara, Ernesto German Cardona-Muñoz, Ernesto Javier Ramírez-Lizardo, Sylvia Elena Totsuka-Sutto, Araceli Castillo-Romero, Teresa Arcelia García-Cobián, Leonel García-Benavides

**Affiliations:** ^1^Instituto de Terapéutica Experimental y Clínica, Departamento de Fisiología, CUCS, Universidad de Guadalajara, Calle Sierra Mojada 950, Colonia Independencia, 44340 Guadalajara, Jal, Mexico; ^2^Laboratorio de Neurofisiología, Departamento de Fisiología, CUCS, Universidad de Guadalajara, Calle Sierra Mojada 950, Colonia Independencia, 44340 Guadalajara, Jal, Mexico

## Abstract

Ischemia/reperfusion (I/R) lesions are a phenomenon that occurs in multiple pathological states and results in a series of events that end in irreparable damage that severely affects the recovery and health of patients. The principal therapeutic approaches include preconditioning, postconditioning, and remote ischemic preconditioning, which when used separately do not have a great impact on patient mortality or prognosis. Oxidative stress is known to contribute to the damage caused by I/R; however, there are no pharmacological approaches to limit or prevent this. Here, we explain the relationship between I/R and the oxidative stress process and describe some pharmacological options that may target oxidative stress-states.

## 1. Introduction

As early as 1986, Murry et al. [1–3] observed that, after occlusion of the coronary artery and posterior reperfusion, lesions were present in myocardial tissue in the dog, which seemed to accelerate necrotic damage. In addition, histopathological changes in tissues observed at 30–60 min of reperfusion were similar to that observed at 24 h of permanent ischemia. Ischemia/reperfusion (I/R) lesions are present in many diseases that affect multiple health systems [4–8]. The effect of these lesions can range from irreversible damage to death of the injured tissue (e.g., cardiovascular, renal, neuronal, and hepatic) [8].

One of the main events that contribute to this damage is the formation of reactive oxidative species (ROS) and reactive nitrogen species (RNS) and subsequent redox signaling disruption in mitochondria [3, 9–11].

The current therapeutic approaches include pharmacological and mechanical interventions. To date, the mechanical approaches (preconditioning, postconditioning, and remote ischemic preconditioning) have proven to be the most promising; however, these methods are invasive, cannot be used for all cases, and the end results can vary. In contrast, the pharmacological interventions currently available are unable to produce any significant effects on patient prognosis [12–20]. The establishment of animal models of I/R injury has aided in determining the molecular mechanisms involved and possible pharmacological targets.

## 2. What Is I/R Lesion?

**Figure 1:**
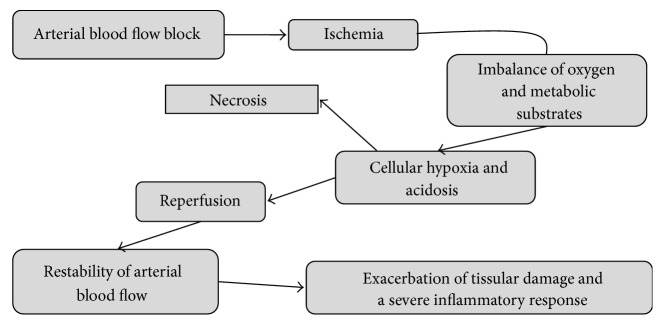
Ischemia reperfusion process. Sequence of stapes and clinical states.

I/R lesions can be defined as a phenomenon that occurs following the block of arterial blood flow to tissue or an organ, which produces a severe imbalance in oxygen and metabolic substrates. This imbalance causes tissue hypoxia and inhibits metabolic processes within cells; paradoxically, the restoration of arterial blood flow and reoxygenation is associated with the exacerbation of tissue damage and a severe inflammatory response (Figure 1) [3, 12–22].

## 3. Mechanism of Lesion

The formation of lesions is caused by multiple events that are triggered by the block of arterial flow and its restoration. The most critical time point in lesion formation is 72 h after reperfusion and the limitation in damage is at 7 days after the initial reperfusion, with recovery taking more than 15 to 90 days [3, 10, 11, 20].

**Figure 2:**
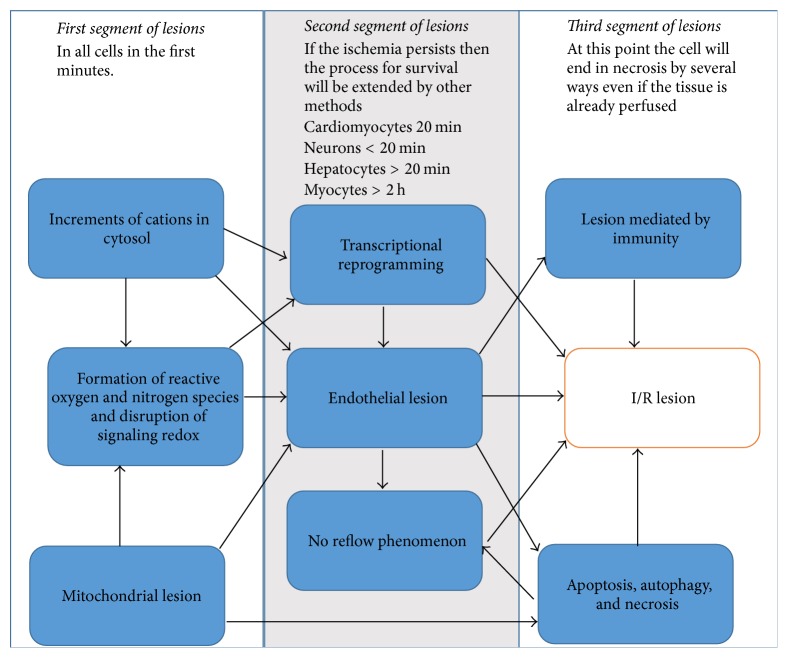
Chronology and correlation of I/R lesion “hot-points.” The ischemic process is distinct in each tissue but can be divided into three segments that are shared among all cell types yet show differences in specific details (e.g., timing). The time to damage is prior point.

There are eight pathophysiological processes that contribute to lesion formation (Figure 2). These processes can act separately and consecutively, and although their order varies depending on the tissue, they all overlap in one crucial step: permanent mitochondrial lesion and redox signaling disruption [3, 10, 11, 21–25].

## 4. Imbalance in Metabolic Substrates and Oxygen

Metabolic substrates such as glucose and oxygen are necessary for mitochondrial ATP production. When oxygen concentration is depleted and aerobic glycolysis stopped, cells switch to anaerobic metabolism, causing an increase in lactic acid production that diminishes cytosolic pH. This acid microenvironment within the cytosol helps cells to survive ischemia. Time is an essential component in reaching this imbalanced state and varies depending on the tissue; for example, cardiac cells can tolerate 20 min of ischemia before necrosis and hepatocytes and renal cells more than 30 min, while neuronal cells can tolerate no more than 20 min. Some tissues (e.g., skeletal muscle cells) excel compared with others and can tolerate 2 h of ischemia [2, 3, 13, 15, 53, 54, 64, 65, 91–106].

## 5. Increase in Cytosolic Cation Levels

During arterial blood flow occlusion (ischemia), cellular metabolism continues. However, the moment an imbalance in cation levels is detected, a series of changes in the cell occurs, which starts with the activation of the Na^+^/Ca^+2^ exchanger and L-type Ca^+2^ channels, which cause an increase in the levels of cytoplasmic Ca^+2^. This increase triggers the activation of Na^+^/H^+^ exchangers that consequently results in an increase in H^+^ and changes the pH of the cell. These events impair the metabolic processes of the cell and affect K^+^ channels in mitochondria [21, 23, 33, 64, 100, 101, 107–115].

During the reperfusion process, the cell tries to restore the change in pH by activating the Na^+^/HCO3^−^ exchanger. The efflux of H^+^ produces an influx of Na^+^ increasing the concentration of Na^+^ in the cytosol. This increase activates the Na^+^/Ca^+2^ exchanger, which leads to an increase in cytosolic Ca^+2^ [21, 23, 33, 64, 100, 101, 107–115]. The high concentration of Ca^+2^ in the cytosol activates several proteases and other proteins that lead to dysfunction and destruction of organelle membranes and corruption of normal metabolism [21, 23, 33, 64, 100, 101, 107–115].

In myocardial cells, changes in membrane potential traffic activity and water migration secondary to voltage-dependent channel aperture lead to arrhythmia and myocardial stunning. In the kidney, glomerular charge is inverted and overloads filtration solutes, increasing water, protein, and electrolyte loss through urine production. In hepatocytes, membrane potential and pH changes are detected, which suppress and activate many enzymes that optimally operate at a neutral pH, and lead to edema and necrosis [21, 23, 33, 64, 100, 101, 107–115].

## 6. Mitochondrial Lesions

**Figure 3:**
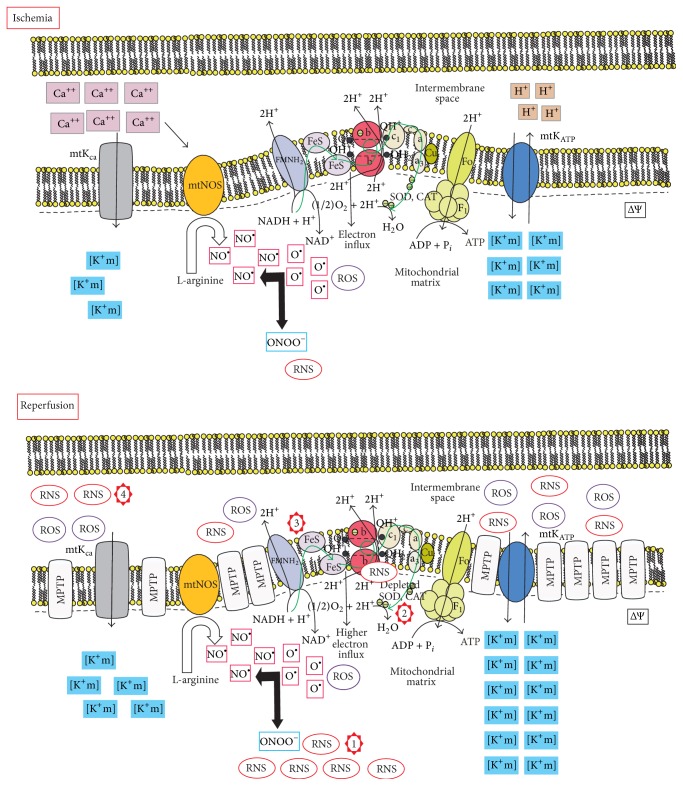
Mitochondrial I/R process. Numbers indicate the four major therapeutic points. (1) Production of ROS and RNS during I/R; (2) depletion of scavenger enzyme systems that reduce free radical; (3) overproduction of superoxide during reperfusion; (4) release and disruption of redox signaling. See the text for a more detailed description on the physiopathological processes.

The metabolic changes in the cytosol following ischemia affect the normal function of mitochondria, which produces an adaptive response brought about by increasing levels of cytosolic Ca^+2^ and decreases in oxygen, NADH, pyruvate, ADP, and Pi. The high concentration of Ca^+2^ activates mitochondrial calcium-sensitive K^+^ channels (mtKca) and mitochondrial nitric oxide synthase (mtNOS), which increases the levels of nitric oxide (NO^•^) (Figure 3) [21, 23, 33, 64, 100, 101, 107–115]. NO^•^ blocks complex IV in the respiratory chain, inducing an influx of electrons into the mitochondrial matrix and depletion of ATP. NO^•^ reduces molecules to superoxide anions (O_2_^−^) and produces high levels of peroxynitrite (ONOO^−^). The loss of ATP is caused by the impairment to ATP recycling, depletion of substrates, and inhibition of complex IV in the respiratory chain [21, 23, 33, 64, 100, 101, 107–116]. This influx of electrons into the mitochondrial matrix and the efflux of protons to the cytosol maintains mitochondrial membrane potential; however, it results in an increase in the production of ROS, RNS, and edema and makes the mitochondrial matrix an alkaline environment [21, 23, 33, 64, 100, 101, 107–116].

The ATP depletion induces the activation of mitochondrial ATP sensitive K^+^ channels (mtK_ATP_), resulting in the influx of K^+^ to the mitochondrial matrix and efflux of H^+^. This change accelerates mitochondrial electron transport in the respiratory chain and produces more influx of electrons to the mitochondrial matrix, which in turn produces more ROS and RNS [21, 23, 33, 64, 100, 101, 107–118].

During reperfusion, the entry of oxygen and metabolic substrates in mitochondria (Figure 3) produces more ROS and RNS. The levels of ROS and RNS and the imminent mitochondrial membrane potential change activate the mitochondrial permeability transition pore (mtPTP), mtK_ATP_, and mtK_Ca_ dissipating membrane potential and releasing all ROS and RNS [21, 23, 33, 64, 100, 101, 107–118].

This is known as the “point of safe return” (PSR): mitochondria have lost their membrane potential, cell activity is dampened until subsequent death, and the I/R lesion has spread to contiguous cells. These contiguous cells attempt to survive injury but enter the I/R lesion state when ROS, RNS, and other molecules are released from the dying cell. It should be noted that the establishment of any therapeutic prevention must take place before PSR, with these strategies known as preconditioning. Any strategies that take place after PSR are known as postconditioning. The PSR process in mitochondria is present in all cells, but the ability to adapt to injury is tissue dependent. This is because of the high levels of oxidative stress (OS) in different tissues, and their mechanisms for adapting to sudden microenvironmental changes [21, 23, 33, 64, 100, 101, 107–118].

## 7. What Is Oxidative Stress?

OS refers to an imbalance between the prooxidant and antioxidant levels, in favor of the prooxidants, in cells and tissues. These changes lead to modification or damage to lipids, proteins, and DNA. Prooxidants cause or promote oxidation. Antioxidants are molecules that inhibit the formation of prooxidants and inhibit oxidation [33, 66, 107, 119–126].

## 8. Formation of Free Radicals

The major source of free radicals in I/R is mitochondria [21, 23, 33, 66, 107, 108, 111, 112, 119–127]. Normally, the electron transport mechanism in the mitochondrial respiratory chain is impaired, this produces ROS from one-electron reduction of oxygen (see (1)) [33, 66, 107, 119–126, 128].

(1) O2→2H+1e−O2−→1e−H2O2→1e−OH•→1e−H2O

is the metabolic process of reduction of free electron. Observe the production of 4 free electrons that must be reduced to water and one diapered electron.

**Figure 4:**
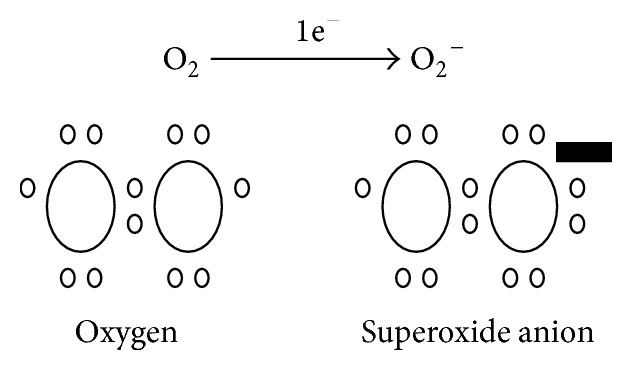
Formation of superoxide anion. The two molecules of oxygen, which are in equilibrated energetic form (6 electrons each one), accept one electron in the last orbital which leads to unstable energy form (7 and 8 electrons), making the molecule of oxygen need to take one electron from the environment to be in its energetic equilibrium form again.

The lifespan of O_2_^−^ (Figure 4) in biological systems is less than a second (50 *µ*s) and has a diffusion distance of ~320 nm. It rapidly reacts with another molecule of superoxide to form hydrogen peroxide (H_2_O_2_) [33, 66, 107, 119–126, 129, 130].

In mitochondria, one of the important reactions is the diffusion reaction between O_2_^−^ with NO^•^ (termed the radical-radical reaction) (see (2)) to form ONOO^−^, which can diffuse across biological membranes at a 400 times greater rate than O_2_^−^. The half-life of ONOO^−^ is <0.1 s and it has high reactivity with organic molecules, especially lipids [33, 66, 107, 119–126, 129, 130].

(2) $$\begin{array}{c} {O_{2}}^{-}+{NO}^{•}⟶{ONOO}^{-} \end{array}$$

is radical to radical reaction to form peroxynitrite on one of the most instable radicals.

Under physiological states, the production of O_2_^−^ produces H_2_O_2_ via manganese superoxide dismutase (MnSOD) in the mitochondrial matrix. This enzyme is found in tetramers, with each subunit consisting of 151 amino acids. MnSOD maintains the steady-state concentration of O_2_^−^ at 10-10 M during acute phases of ischemia, but when this phase is over, the activity of MnSOD increases, which results in the production of massive levels of O_2_^−^ that are reduced by NO^•^. Because of membrane potential, this molecule stays in the mitochondrial matrix. During reperfusion, the respiratory chain accelerates and O_2_^−^ is overproduced. MnSOD competes with NO^•^ to reduce the amount of O_2_^−^ and consequently forms more ONOO^−^, which is released into the cytoplasm via the mtPTP [33, 66, 107, 119–126, 129, 130].

## 9. How Free Radicals Can Cause Damage to Cells?

The free radicals “take” one electron from the adjacent molecule, which leads to the formation of a new free radical that will “take” one electron from the adjacent molecule. Therefore, a chain reaction occurs that will only end when the free radicals are reduced by antioxidants [25, 123, 125, 126, 128, 130].

## 10. Damage to Lipids in I/R

The interaction between OS and lipids is one of the most prevalent causes of cellular injury. The degradation products of lipid peroxidation are aldehydes, such as malondialdehyde (MDA), 4-hydroxynonenal (4-HNE), and hydrocarbons such ethane and ethylene. Lipid peroxidation in mitochondria is particularly cytotoxic and has multiple effects on enzyme activity and ATP production, as well as on the initiation of apoptosis [107, 110, 115, 116, 119, 122–125, 130].

## 11. Damage to Proteins in I/R

Damage to proteins occurs through site specific amino acid modifications, fragmentation of the peptide chain, aggregation of cross-linked reaction products, altered electrical charges, and increased susceptibility to removal and degradation. The activity of ONOO^−^ produces nitrotyrosine; meanwhile O_2_^−^ inactivates enzymatic function [107, 110, 115, 116, 119, 122–125, 130].

## 12. Damage to DNA in I/R

OS can induce numerous lesions in DNA that cause deletions, mutations, and other lethal genetic effects. The sugar and the base fraction are susceptible to oxidation causing base degradation, single-strand breakage, and cross-linking to proteins. One product of DNA damage is 8-oxo-7,8-dihydro-2′-deoxyguanosine (8-Oxo-dG) [107, 110, 115, 116, 119, 122–125, 130].

## 13. Pathways Affected by Redox Signaling in I/R

Redox signaling describes the action of ROS and RNS on altering intrinsic cellular activity. At low concentrations ROS and RNS work as signaling molecules, while at high concentrations they damage multiple structures, especially mitochondria [23–25, 67, 119, 122, 129, 131].

The mechanism by which OS alters protein function and structure involves redox-reactive cysteine residues on proteins. Oxidation of these residues forms reactive sulfenic acid, which in turn forms disulphide bonds with nearby cysteines, and undergoes further oxidation into sulfinic or sulfonic acid, or sulfenamide when nitrogen is present locally. These redox modifications are reversible through reducing systems such as thioredoxin and peroxiredoxin [23–25, 67, 119, 122, 129, 131].

## 14. The Mitogen-Activated Protein Kinase Cascade in I/R

The mitogen-activated protein kinase (MAPK) cascade consists of a three-rung kinase tier. The canonical cascade occurs when MAPK kinase kinases (MAPKKK) phosphorylate and activate MAPK kinases (MAPKK), which phosphorylate and activate MAPKs [23, 132–139].

There are two noncanonical pathways: the apoptosis signal-regulated kinase 1 (ASK1) pathway and cGMP-dependent protein kinase (PKG) pathway. In the former MAPK cascade, ASK1 is an upstream MAPKKK that regulates c-Jun N-terminal kinases (JNK) and p38 kinase (p38), leading to apoptosis via phosphorylation of MKK4, MKK3, and MKK6 MAPKKs. ASK1 is activated by OS and mediates p38 signaling, which leads to differentiation and immune signaling. However, activation of ASK1 by high levels of OS (via oxidization of two cysteine residues in the redox center of thioredoxin) induces ASK1 dissociation and allows its complete oligomerization with tumor necrosis factor-*α* receptor associated factor (TRAF) and ASK2, thereby promoting cell death. Alternatively, the PKG pathway is integrated by PKG1*α*, protein kinase A (PKA), and protein kinase C (PKC) and, similarly, is also regulated by a redox mechanism [23, 132–139].

## 15. The Phosphoinositide 3-Kinase Signaling Pathway in I/R

Phosphoinositide 3-kinase (PI3K) consists of one catalytic (p110) and one regulatory (p85) subunit and is firmly coupled with the receptor tyrosine kinase (RTK) family, which are activated by several growth factors. PI3K catalyzes phosphatidylinositol 4,5-diphosphate (PIP2) to synthesize the second messenger, phosphatidylinositol 3,4,5-triphosphate (PIP3). PIP3 serves as a membrane-bound signaling molecule that recruits proteins containing pleckstrin homology (PH) domains, such as phosphoinositide-dependent protein kinase (PDK) and protein kinase B (AKT) serine threonine/kinase, which mediate further downstream signaling events. Phosphatase and tensin homolog (PTEN) phosphorylates PIP3, causing inhibition and ensuring that the PI3K pathway is subject to reversible redox regulation by OS. Oxidation of PTEN by OS leads to persistent activation of the PI3K pathway, causing permanent activation of RTKs [14, 19, 134, 140–146].

## 16. The Redox Factor-1 and NF-E2-Like 2 (Nuclear Factor Erythroid 2) Pathway in I/R

Redox factor-1 (Ref-1) is a multifunctional protein that regulates transcription factor activity and also mediates base excision repair. The transcriptional regulatory function of Ref-1 is exerted by its redox activity on several transcription factors, including activator protein 1 (AP-1), p53, nuclear factor kappa B (NF-*κ*B), and hypoxia inducible factor 1 (HIF-1).

Ref-1 activates the AP-1-Fos-Jun complex via redox regulation of cysteine residues in Fos-Jun DNA binding domains. As it mediates both DNA repair and redox activation of key transcription factors involved in cellular defense (including AP-1 and NF-*κ*B), upregulated Ref-1 activity protects DNA from oxidative damage. Consequently, OS conditions can activate detoxification genes such as glutathione S-transferase (GST), NADPH quinone oxidoreductase-1 (NQO1), heme oxygenase-1 (HO1), and ferritin H (FH) [23–25, 67, 119, 122, 129, 131].

NF-E2-like 2 (Nrf2) is another transcription factor, which activates antioxidant responsive element- (ARE-) dependent transcription of target genes under OS. These genes serve as antioxidants in processes such as electrophile detoxification, glutathione synthesis, and ROS homeostasis [23–25, 67, 119, 122, 129, 131, 147].

The interaction between these two pathways has a protective, synergistic effect against OS. During I/R, Ref-1 and Nrf2 upregulation increases expression of NF-*κ*B, which increases apoptosis and inflammation [23–25, 67, 119, 122, 129, 131, 147, 148].

## 17. Transcriptional Reprograming in I/R 

The alteration in transcriptional control of gene expression induced by I/R lesions is known as transcriptional reprograming. Oxygen depletion and increased ROS, RNS, and apoptosomes lead to transcriptional alterations within the nucleus. Damage (secondary to redox signaling disruption) causes toll-like receptor (TLR) expression in the membrane of affected cells, indirectly affects NF-*κ*B expression, and increases MAPK and interferon activity. TLR3 is overexpressed in necrotic cells, TLR2 is overexpressed in hypoxic and inflammatory states, while TLR4 is exclusively overexpressed in renal I/R lesions. During the process of adaption, cells affected by I/R lesions show specific expression of micro-RNAs (miRNAs), which modulate gene expression through transcriptional and posttranscriptional pathways. For example, miRNA-21 blocks PTEN expression in the ischemic state and reduces apoptosis in the first 48 h but shows persistent overexpression during this time and subsequently increases apoptosis as a result of PTEN reduction. Moreover, miRNA-378 blocks caspase-3 expression and reduces apoptosis in the reperfusion state. Expression of multiple genes depends on the amount of OS, time of ischemia, and number of necrotic cells produced during ischemia [10, 11, 19, 22, 23, 93, 98, 132–139, 149–170].

## 18. Apoptosis, Autophagy, and Necrosis in I/R

The process of cellular destruction starts after mitochondrial lesion, disruption of redox signaling, and transcriptional reprograming. During apoptosis and after PSR is reached, the mtPTP releases cytochrome C from the membrane, activating the caspase cascade and pannexin hemichannels, which release ATP and work as beacons for phagocytic cells. Redox signaling disruption and transcriptional reprograming lead to NF-*κ*B activation, which activates apoptosis and is histologically characterized by nuclear fragmentation, endosome formation, mitochondrial and cellular contraction, and loss of membrane potential. Autophagy is also produced as an adaptive response to sublethal OS, with metabolic change and transcriptional survival reprograming in endosomes, loss of organelles, and formation of coil-shaped vacuoles observed. Cell damage can progress to necrosis, with or without the presence of edema within cells and organelles, or cause cell membrane disruption and efflux of enzymes to the extracellular space [10, 11, 19, 22, 23, 93, 98, 132–139, 149–176].

## 19. Immunity-Mediated Lesions in I/R

During the I/R lesion, three pathways are activated: sterile inflammation, adaptive response, and innate autoimmunity. Sterile inflammation is mediated by TLRs, which lead to NF-*κ*B, MAPK, and interferon activation. These receptors also produce chemotaxis of inflammatory and phagocytic cells, which start the inflammatory response. After 24 h of reperfusion, the adaptive response begins, with expansion and recruitment of T cells. At 72 h, the highest level of response and T_reg_ depletion is reached. This affects the innate response, leading to autoimmunity characterized by autoantibody formation by B cells, complement activation, and Bcl3 depletion, which inhibits granulopoiesis production [10, 11, 22, 97, 152, 177–180].

## 20. Endothelial Lesion

Endothelial lesions that present during the ischemic state are principally affected by decreased oxygen, increased ROS and RNS, and redox signaling disruption, followed by membrane potential loss and increasing membrane permeability, chemotaxis, and imbalanced capillary vasoconstriction/vasodilatation factors. In the reperfusion state, the endothelium suffers lesions caused by the immune response and activation of the coagulation system, increasing leucocitary adherence, and platelet-leucocitary interaction. This process is produced by mechanical brushing that occurs in the damaged endothelium and causes e-selectin adherence protein expression in the membrane, which interacts with the ligand, selectin-1, and in neutrophils coactivates integrin-*αμβ*2. Following interaction and formation of this complex, neutrophils are able to bind to erythrocytes and platelets through their membrane, which directs them to the damaged tissue and increases the inflammatory response in affected tissue [10, 11, 22, 117, 127, 180, 181].

## 21. No Reflow Phenomenon

The no reflow phenomenon is present in the reperfusion state because of endothelial cell injury, activation of the coagulation process, and increased leucocitary adherence. This phenomenon increases impedance of microvascular flow and capillary occlusion by leucocytes and is present in 60%–68% of all I/R cases [9–11, 22, 91, 182].

## 22. Analysis of the Pharmacological Approach in I/R

The actual pharmacological approach to prevent or mitigate I/R lesions has so far been unsuccessful. This is likely because one drug cannot cure all disturbances, and, indeed, the phenomenon is a result of several events that follow a specific sequence. Consequently, attempting to cover the physiopathological process with just one medication is likely not enough. Most studies have attempted to show that one drug will make a difference, and regarding I/R lesion in vitro experiments and preclinical studies have shown good results. Nonetheless, the lesion is manifested systemically and affects several processes; therefore, the correct approach needs several drugs targeting the physiopathological process (Figure 3) [1–3, 55, 64, 65, 75, 92, 94, 95, 100, 104, 183–185].

## 23. The Gender Aspect of I/R

Recent studies dealing with survival in specific pathological conditions related to I/R have shown significantly better patient survival in females than males. However, other studies have shown a much poorer outcome associated with female patients. The reason for these controversial results is not clear, but a variety of different factors may influence interpretation of these studies. For example, age (postmenopausal), race, underlying disease, and/or medications may impact the outcome. Failure to account for and control these different variables makes it difficult to accurately assess the role of gender in reducing or increasing survival expectancy following I/R lesions [186–191].

## 24. The Physiopathological Approach

The critical points in OS in I/R are the production of prooxidants, the depletion of SOD, the accumulation of free radicals, loss of mitochondrial membrane potential with subsequent release from mitochondria, and redox signaling disruption. There are several drugs that may have therapeutic potential (Table 1).

## 25. Electron Acceptor

**Figure 5:**
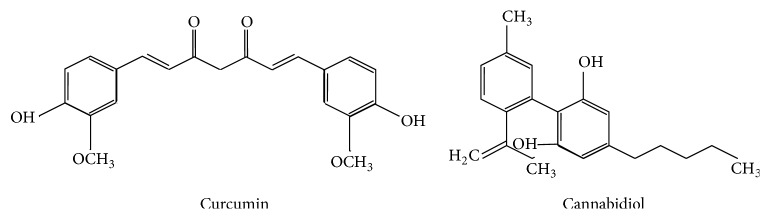
Structure of curcumin and cannabidiol, with properties to interact with free radicals in their proximity.

The process of reducing free radicals is made possible based on the structure of the molecules. These drugs are equipped by one or more aromatic ring with hydroxyl groups in their structures. Therefore, they can exchange the free radical with the hydroxyl group and end the chain reaction of free radical accumulation. The structure of curcumin and cannabidiol (Figure 5) has a typical antioxidant architecture that confers no enzymatic scavenging ability [24, 26–29, 33–35]. Besides, the cannabidiol in the liver and cardiac I/R lesion has shown that it could interact directly with the cannabinoid CB_2_ receptor. In the heart the agonism of the CB_2_ receptor was shown to modulate the myocardial inflammation and attenuates the infarct size (142) and decreases the myocardial ROS and RNS generation, restores the glutathione content and SOD activity, and modulates the signaling redox and the NF-*κ*B activation (143). In the liver the cannabidiol attenuates tissue oxidative and nitrative stress, acute and chronic hepatic inflammatory response, signaling redox, and cell death by its strong antioxidant ability and the interaction with the CB_2_ receptor (144).

## 26. Block of Mitochondrial Respiratory Chain Complex I

Complex I in the mitochondrial respiratory chain is the most important for the production of O_2_^−^, which is produced by the reverse transport of electrons from complex II. During the ischemic phase, the partial disablement of this primary component results in free radical formation because of the strain it has on the electron transport to complex III, and, during the reperfusion phase, the production of O_2_^−^ is attenuated [36–38, 44]. One of the actions of complex I inhibitor metformin is to induce mild and specific inhibition of mitochondrial respiratory chain complex I, which reduces free radical formation [44].

## 27. Peroxisome Proliferator Activated Receptor-***γ*** (PPAR-***γ***) Agonist

This class II nuclear receptor interacts with multiple survivor genes and can down- or upregulate proteins involved in the tolerance to I/R injury. Normally, PPAR-*γ* works as a heterodimer with retinal receptor but interacts with multiple response systems in the DNA; activation of this receptor can increase the expression of MnSOD and other enzymatic scavengers and blocks the induction of apoptosis. The PPAR-*γ* agonists pioglitazone and telmisartan increase the bioavailability and action of PPAR-*γ* to improve cell survival [45–47, 53–55, 76–78, 192–194].

## 28. Pleiotropic Effects

There are multiple pathways involved in the production of OS that can be modulated, and several in vitro studies and animal models have shown promising results. In vitro experiments using atorvastatin have shown a reduction in ROS and RNS levels in I/R and various injury models including degenerative pathologies and chronic diseases. In addition, atorvastatin was shown to activate nuclear receptors such PPAR-*γ*. Telmisartan has a similar effect and reduces OS by activating PPAR-*γ*, blocking the angiotensin II receptor, type 1 (AT1 receptor), increasing levels of enzymatic scavengers, and activating cell survival pathways [45–47, 53–58, 65–68, 76–78, 192–194].

## 29. Prejudice in the Physiopathological Approach

The adverse effect of drugs may influence the therapeutic effect, with possibly the most questionable drugs being atorvastatin (statins) and pioglitazone (PPAR-*γ* agonist). The atorvastatin controversy owes to the associated increased risk of diabetes mellitus, with a meta-analysis showing that, after at least 4 years of treatment, patients have an incidence of 5%-6% for diabetes mellitus onset and decrease in serum ubiquinone levels of 32%–54% during statin use for at least three months. However, treatment for I/R lesions last for only a short period of time and should be administered during the first hours of diagnosis and for not longer than two weeks, to reduce the chance of suffering major adverse effects. It is well known that pioglitazone should not be used in class II or III New York Heart Association heart failure scale patients, or those with a depressed ejection fraction of less than 40%, due to exacerbation of congestive heart failure (approximately 9%) in studies when the treatment time lasted from 3 weeks to 3 months using the maximal recommended dose. Treatment with pioglitazone for I/R lesions should not surpass the recommended dose or last longer than two weeks, as mentioned above.

## 30. Future Prospects

Several attempts have been made to inhibit I/R lesions, but the real challenge lies in attenuating the processes that lead to the formation of lesions, which include the mitochondrial production of ROS and RNS and disruption of signaling redox. To date, most research has focused on the inflammatory response [96, 97], and there is limited knowledge on the effect of preconditioning, postconditioning, and remote ischemic preconditioning. The most studied therapeutic approaches with respect to I/R lesions are the mechanical process of preconditioning, postconditioning, and remote preconditioning [1, 2, 10, 12, 13, 15–18, 20, 21, 64, 92, 93, 108, 109, 114, 118, 142, 195–204]; however, the results are controversial, and the greatest benefits have only been observed in animal models [2, 10, 64, 197]. In addition, meta-analysis and randomized trials have shown that this procedure has no beneficial effect on mortality but can improve periprocedural myocardial infarction and afford some neuroprotection [196, 203, 204]. However, it should be noted that this procedure needs to be performed within a strict timeframe and is dependent on the condition of the patient. Therefore, not all patients are suitable candidates. To complicate matters, the heterogeneity of injury also limits the effectiveness of this method. These variables have biased the statistical evaluation of this approach. Nevertheless, no one has implemented a pharmacological regimen for the management of OS, before, during, or after the procedure despite all the evidence for the involvement of OS.

## 31. Conclusion

OS in I/R lesions has a big impact on the activation of multiple secondary mechanisms of damage. Therefore, the search for a therapeutic pharmacological regimen that can inhibit the production of ROS and RNS and can modulate the signaling redox should take priority in the treatment of I/R lesions. There are currently no studies on pharmacological regimens at any institution or clinical trials for patients who suffer the I/R phenomenon in combination with other strategies of mechanical procedure. The establishment of new animal models and studies focusing on the mechanism of action of known drugs that have been used for other pathological states has revealed that reducing OS can provide beneficial outcomes. Perhaps combination therapy could attenuate OS further and provide a better prognosis for patients who suffer from this phenomenon following mechanical procedures. There is no sufficient data to confirm or reject this hypothesis. Therefore, more studies should be performed to determine a standardized therapeutic regimen to control or prevent the OS damage in I/R before, during, and after mechanical procedures.

## Figures and Tables

**Table 1:** Promising drugs in the I/R lesion.

Drug	Molecular mechanism	Beneficial effect in IR	Adverse effects	Organ cell major effect	Literature support study
Curcumin	Electron acceptor	Reduction of free radicals	Dermatitis Bitter taste	Neurons	[26–32]
Cannabidiol	Electron acceptor	Reduction of free radicals, modulation of inflammation and signaling redox	Neuronal disorders	Neurons Hepatocyte Cardiomyocyte	[24, 33–43]
Metformin	Block of complex I in respiratory chain	Modulated production of free radicals	Lactic acidosis Blood coagulation disorder Liver function test abnormal Encephalopathy	Hepatocyte Cardiomyocyte Neurons	[44–52]
Pioglitazone	^*∗*^PPAR-*γ* agonist	Increments on the expression of ^*∗∗*^MnSOD and some other survival gens	Congestive heart failure Edema Liver function test abnormal Osteopenia	Endothelial cells Cardiomyocyte Hepatocyte	[50–63]
Atorvastatin	Pleiotropic effects	Reduction of free radicals, increments on expression of MnSOD, modulation of survival gens	Diabetes mellitus Reduction of ubiquinone level Liver function test abnormal Autoimmune disease Rhabdomyolysis Acute kidney injury	Endothelial cells Cardiomyocyte Hepatocyte Musculoskeletal	[30–32, 64–74]
Telmisartan	Pleiotropic effects	Reduction of free radicals, increments on expression of MnSOD, modulation of survival gens	Angina pectoris Edema Carcinogen effect Liver function test abnormal	Endothelial cells Cardiomyocyte Hepatocyte	[58, 75–90]

^*∗*^PPAR-*γ*: peroxisome proliferator activated receptor-*γ*. ^*∗∗*^MnSOD: manganese superoxide dismutase.
